# White matter hyperintensity mediating gait disorders in iNPH patients *via* neurofilament light chain

**DOI:** 10.3389/fnagi.2023.1117675

**Published:** 2023-03-16

**Authors:** Lu Yang, Fuxia Yang, Yao Deng, Aijuan Yan, Wenshi Wei, Xuhao Fang

**Affiliations:** ^1^Department of Neurology, Huadong Hospital Affiliated to Fudan University, Shanghai, China; ^2^Department of Neurosurgery, Huadong Hospital Affiliated to Fudan University, Shanghai, China

**Keywords:** white matter hyperintensities, idiopathic normal pressure hydrocephalus, gait disorder, cerebrospinal fluid, nerve filament light

## Abstract

**Purpose:**

This study aimed to analyze the differences in regional white matter hyperintensities (WMH) volume and cerebrospinal fluid biomarker levels between idiopathic normal pressure hydrocephalus (iNPH) patients with or without gait disorder.

**Methods:**

Forty-eight iNPH patients undergoing bypass surgery and 20 normal senile individuals were included. The LST toolkit was used to segment all MRI fluid attenuation inversion images and quantify the WMH volume in each brain region. Cerebrospinal fluid was collected from all individuals and measured for concentrations of Aβ, t-tau, p-tau, and neurofilament light chain (NfL). Patients with iNPH were followed up for 1 year and divided categorized into a gait disorder improvement group and no improvement group according to the 3 m round-trip test time parameter improvement by more than 10%.

**Results:**

We found that WMH in all areas of iNPH patients was higher than that in the control group. CSF levels of Aβ, t-tau, and p-tau were lower than those in the control group, while NfL levels were higher than those in the control group. The gait (+) group NfL level was higher than that in gait (−), and there were no statistical differences in Aβ, t-tau, and p-tau levels. The gait (+) group of frontal and parietal lobe WMH volume PVH above the gait (−) group. The mediating effect model analysis showed that PVH might affect the gait disorder of iNPH patients through NfL. A 1-year follow-up of the patients after the bypass surgery found that 24 of the 35 patients in the gait (+) group had improvements, while 11 had no significant improvements. The comparison of CSF marker levels between the two groups showed that the CSF NfL level in the improved group was lower than that in the non-improved group. The WMH volume and PVH in the frontal–parietal lobe of the improved group were lower than those of the non-improved group.

**Conclusion:**

iNPH patients have more serious frontoparietal and periventricular white matter lesions, and WMH volume in the frontoparietal may mediate the occurrence of gait disorder in iNPH patients through the increase of NfL level.

## 1. Introduction

About 50 million people worldwide currently have dementia, which is expected to triple by 2050, reflecting a rapidly aging population, and of those 50 million people with dementia, about 10 percent have idiopathic normal pressure hydrocephalus (iNPH) (Torretta et al., 2021), potentially reversible dementia with a clinical triad of gait disorder, urinary incontinence, and dementia (Mori et al., 2012). The clinical manifestations and imaging of iNPH easily overlap and are confused with many common diseases in the elderly. It is very challenging to diagnose and select surgical indications correctly. The pathogenesis and pathophysiology of iNPH are still not well understood. The current consensus is that ventricle enlargement induced by cerebrospinal fluid (CSF) dynamics may trigger a vicious cycle of iNPH nerve damage. Pathophysiological factors such as hypoperfusion, lymphatic injury, metabolic disorders, astrogliosis, neuroinflammation, and destruction of the blood–brain barrier jointly cause white matter and gray matter lesions, ultimately leading to various clinical symptoms of iNPH (Bateman, 2008; Wang et al., 2020).

Leukoencephalopathy is widespread in iNPH patients (Tisell et al., 2011; Fällmar et al., 2021), and its imaging features are difficult to distinguish from ischemic leukoencephalopathy. White matter lesions of iNPH are characterized by hypersignal on T2-fluid attenuated inversion recovery (FLAIR) MRI sequences, especially periventricular white matter hyperintensity (PVH), mainly in the frontal and occipital areas (Tullberg et al., 2007). Studies have shown that PVH irregularly is associated with great vascular diseases (such as aortic and carotid atherosclerosis) (de Leeuw et al., 2000a,b), which may be the result of chronic hemodynamic dysfunction with ischemic demyelination and axon loss (Román, 1987; van Swieten et al., 1991). Deep white matter hyperintensity (DWMH) is associated with vascular complications such as hypertension and lacunar infarction (Mäntylä et al., 1999; Pantoni et al., 2005; Doubal et al., 2010), and it is generally believed that these peripheral lesions represent ischemic tissue damage secondary to brain small vessel disease. Some studies believe lateral ventricular and deep white matter lesions reduce brain plasticity, which is negatively correlated with the surgical effect (Krauss et al., 1996). Therefore, for patients with iNPH combined with leukoencephalopathy, the choice of surgery is often very careful. However, it can be observed in our clinical work that the clinical symptoms of iNPH patients can be significantly relieved by cerebrospinal fluid drainage surgery, accompanied by the obvious absorption of some leukoencephalopathy. Other studies have come to the opposite conclusion.

Symptoms of iNPH can be attributed partly to white matter compression and stretching. When the motor nerve fibers of the corticospinal tract are pulled, gait disorders may occur (Gleason et al., 1993). Pulling the sacral fibers of the corticospinal tract may disrupt bladder contraction and lead to urinary incontinence (Basser and Jones, 2002). Gait and balance disorder is the core symptom of iNPH patients, which is a high-grade frontal lobe gait disorder characterized by a small and unbalanced gait (Morel et al., 2019). Although this movement disorder can be improved by performing a cerebrospinal fluid release test or a shunt, this invasive procedure has an uncertain reactivity. It can cause complications such as infection, and a clearer understanding of the neurophysiological mechanisms may provide a new approach to response prediction. The gait disorder appeared early or initially, and most patients improved significantly after the lumbar puncture drainage test and shunt, which was easy to observe. Therefore, early identification, diagnosis, and intervention are necessary for clinical practice.

In the neurodegenerative diseases field, we usually use some cerebrospinal fluid-specific biological criteria for neurodegenerative diseases, which are usually used for diagnosis and prediction. Current studies have shown AD-related core pathological changes in iNPH, including amyloid deposition (Agren-Wilsson et al., 2007; Hamilton et al., 2010; Leinonen et al., 2011) and dysregulation of t-tau and p-tau (Kudo et al., 2000). However, the correlation between more extensive AD biomarkers and postoperative clinical features of iNPH is inconclusive (Hannun and Obeid, 2018; Jeppsson et al., 2019). NfL is a scaffold protein of the neuronal cytoskeleton, which is elevated in several neurodegenerative diseases and is presumed to leak into cerebrospinal fluid during axonal injury and is a general biomarker of neurodegeneration (Bridel et al., 2019). The role of the axonal biomarker NfL as a predictor of symptoms in patients with iNPH has also not been extensively studied. Tullberg et al. found a six-fold increase in NfL in NPH (Normal pressure hydrocephalus) patients compared with healthy age-matched controls and a correlation between high levels of NfL and gait disorders, urinary incontinence, neuropsychological assessment, and social dysfunction (Tullberg et al., 1998). Other studies have shown that the level of cerebrospinal fluid NfL in NPH patients is increased, which is related to the degree of PVH lesions. The more postoperative NfL decreased in NPH patients, the greater the degree of PVH recovery and the more significant the overall postoperative improvement (Tullberg et al., 2007).

In summary, we know that leukoencephalopathy is widespread in patients with iNPH and is correlated with the clinical symptoms of patients. There are also changes in characteristic markers in the cerebrospinal fluid of iNPH patients, which can be used to diagnose and predict the outcome after shunt surgery. Therefore, we proposed a study on the relationship between the degree of leukoencephalopathy in various areas and cerebrospinal fluid markers and gait disorders in patients with iNPH.

## 2. Materials and methods

### 2.1. Participants

iNPH patients were continuously collected in the neurosurgery ward of Huadong Hospital, Affiliated with Fudan University, from January 2019 to December 2021(>60 year old). ① Inclusion criteria: patients met the diagnostic criteria of the third edition of iNPH guidelines (Nakajima et al., 2021). Clinically, there is at least one symptom of the triad of typical gait disorder, cognitive dysfunction, and urinary incontinence, and the symptoms persist for more than 6 months; Imaging showed ventricle enlargement (Evan’s index >0.3) and no other causes of ventricle enlargement. There may or may not be signs of low density (CT) or high signal (T2-weighted image of MRI) around the ventricle; Coronal image showed “DESH”; Lumbar puncture (lateral decubitus) or intraventricular ICP monitoring confirmed that ICP ≤ 200mmH2O, routine and biochemical examination of cerebrospinal fluid was normal. The lumbar puncture discharge test was positive; Clinical, imaging, and biochemical examinations ruled out the presence of other neurological and non-neurological disorders that might have caused the above clinical manifestations. ② Exclusion criteria: did not meet the diagnostic criteria of the third edition of iNPH guidelines; Absolute contraindications exist; Patients and family members do not want to participate in the study; Patients with secondary NPH secondary to traumatic brain injury, subarachnoid hemorrhage, intracranial infection and other primary diseases were excluded. Patients treated by third ventriculostomy, lateral ventricle Ommaya sac implantation, extracellular ventricular drainage, and other surgical methods were excluded—patients with AD or vascular dementia, Parkinson’s disease, and other neurodegenerative diseases. Twenty healthy elderly patients undergoing hemorrhoid surgery were collected in the inpatient department of anorectal surgery: ① There were no symptoms of gait disorder and cognitive impairment. ② Head MRI imaging showed no ventricular enlargement or other acute lesions. ③ No other systemic diseases in internal medicine.

### 2.2. Clinical evaluation

Gait assessment consisted of a 10-meter straight walk and a 3 m-time up & go (TUG). 10 m straight walk test: The time and number of steps required for 10 m straight walk were measured according to the daily walking state or auxiliary state. After the lumbar puncture drainage test or shunt surgery, if one parameter improved by more than 20% or both of the two parameters improved by more than 10%, it was considered positive. 3 m-TUG: The 3 m round trip test: The patient sits in an armchair with his back resting on the back of the chair and his hand resting on the arm of the chair. After walking to a line 3 m away, the patient turned and returned to the chair to sit down. The test ends when the patient’s hips touch the seat. Patients should be instructed to use a comfortable and safe walking pace during the procedure. Appropriate AIDS can be given. The time and number of steps required for the patient’s trial were recorded. Improvement of more than 10% in both parameters was positive.

In addition, we used the iNPH grading scale (iNPHGS) scale to establish a comprehensive functional evaluation, in which an increase of more than 3 points on the iNPHGS scale was a positive result. According to the gait score in iNPHGS scale: (1) 0, normal; 1 score, there is the chief complaint of vertigo and walking difficulty statement, but no objective gait disorder; (2) 2 points, unstable but can walk independently; (3) 3 points, can walk with the aid of any foreign objects; and (4) 4, unable to walk. The score above 0 was recorded as the gait disorder group, and 0 was recorded as the non-gait disorder group.

For the lumbar puncture drainage test, it is recommended to release 30–50 mL of CSF each time in a single lumbar puncture drainage test. When the CSF release is insufficient to meet the above standards, the final pressure of lumbar puncture 0 is the termination point. MMSE assessment was performed before and 24–48 h after infusion, and an increase of more than 3 points in MMSE score was positive. ICIQ-IF urinary incontinence assessment Scale can be based on the questionnaire form to ask patients and caregivers, according to the severity and frequency of the score.

**Figure 1:**
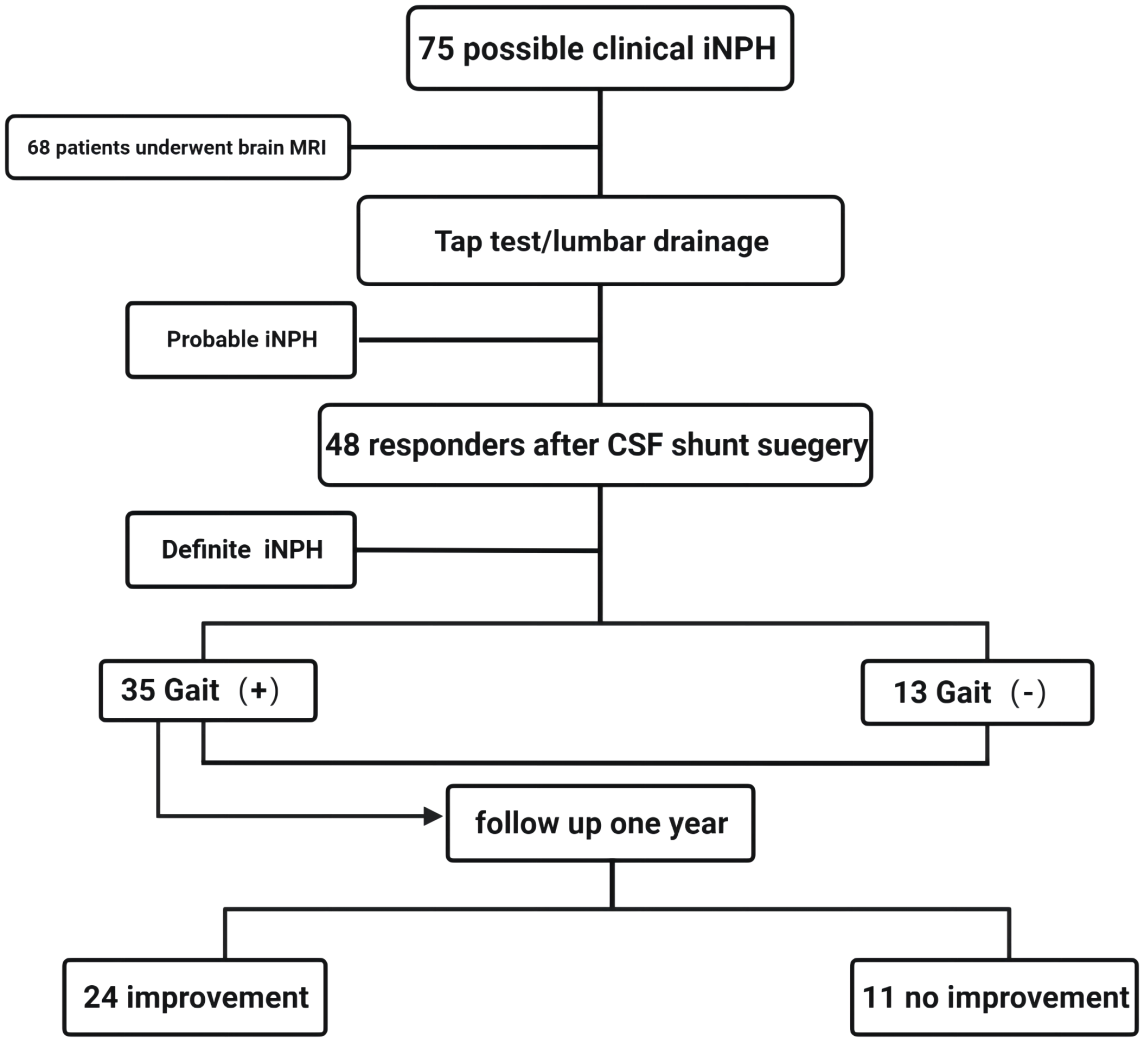
Flow chart of iNPH patient inclusion, evaluation, and follow-up.

Cerebrospinal fluid shunt surgery was performed on all patients with positive lumbar puncture drainage tests, and the positive patients were diagnosed as iNPH patients after postoperative evaluation. The patients were followed up for 1 year after surgery. The above contents were repeatedly evaluated, and the patients were further grouped according to whether the gait disorder improved after 1 year. The patients were defined as follows according to whether there was improvement in their gait disorder after 1 year: the patients with more than 10% improvement in time and gait parameters were classified as the improvement group in the 3 m-TUG, and the rest were the no improvement group. Figure 1 shows the flow of iNPH patient data collection.

### 2.3. Cerebrospinal fluid collection and treatment

After the relevant informed consent was signed, CSF samples were collected by lumbar puncture before shunt surgery, placed in a 15 ml polypropylene tube, and centrifuged at 4,000 rpm and 4°C for 10 min. If the supernatant was blood, the patient was excluded from the study. The supernatant of the centrifuged CSF was categorized into 1 mL of 1.5-mL polypropylene tubes and stored at −80°C. Quantitative analyses of cerebrospinal fluid markers were performed by commercial laboratory partners and classified according to standardized cut-off values (MVZ Synlab Leverkusen, Leverkusen, Germany). Standardized sandwich ELISA methods were used to measure core biomarkers, namely INNOTEST^®^-Aβ42, INNOTEST^®^t-tau, and p-tau. Hypersensitive multifactor electrochemiluminescence (MSD) method to detect Neurofilament Light.

### 2.4. Magnetic resonance imaging acquisition and processing

**Figure 2:**
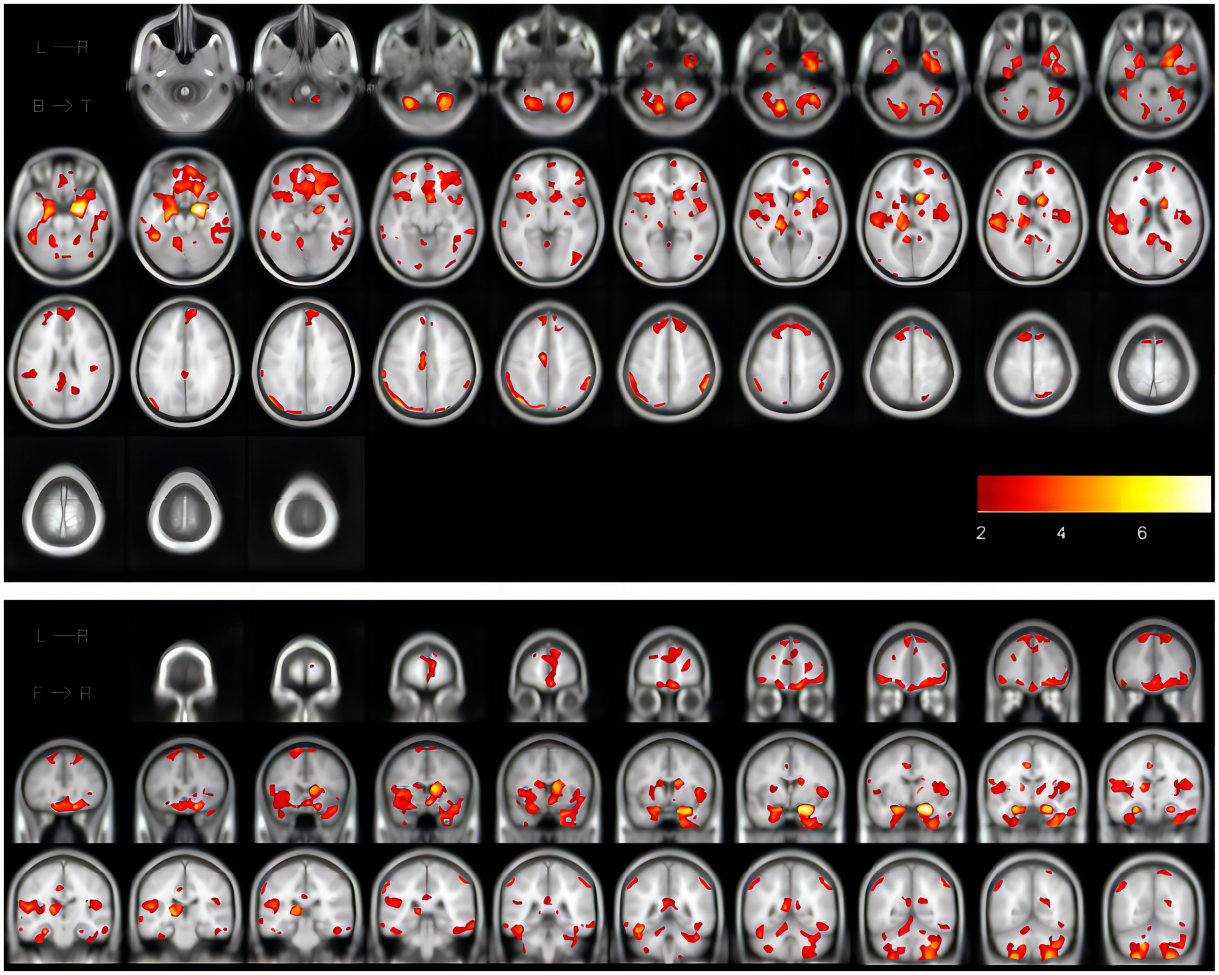
The coronal and cross-sectional images showing the results of white matter segmentation of one iNPH patient using the LST toolbox, in which the WMH is the marked yellow area in the figure. iNPH, idiopathic normal pressure hydrocephalus; LST, Lesion Segmentation Tool; WMH, white matter hyperintensities.

All enrolled patients underwent routine head MRI scanning by MAGNETON Skyra3.0 T magnetic resonance scanner in the Radiology Department of Shanghai Huadong Hospital. These include high-resolution longitudinal relaxation-weighted images (T1WI), transverse relaxation-weighted images (T2WI), diffusion-weighted images (DWI), and Fluid attenuated inversion recovery (FLAIR). Scanning parameters: T1WI: repeat time/echo time (TR/TE) = 220/2.46 ms, section thickness = 5 mm, spacing =1.0 mm, FOV = 23.00 cm; T2WI: TR/TE = 4000/92 ms, section thickness = 5 mm, FOV = 24.00 cm; flair: TR/TE = 7,000/85 ms, slice thickness = 5 mm, FOV = 23.00 cm; DWI: TR/TE = 1,300/62 ms, section thickness = 5 mm, FOV = 24.00 cm. In visual analysis, WMH was identified as a lesion with a spotty or diffuse area showing a high signal on T2W and FLAIR images. WMH was categorized into each lobe’s WMH, deep white matter hyperintensity (DWMH), and periventricular white matter hyperintensity (PVH). We performed this by running the disease segmentation Tool 2.0.15 version based on SPM12 (Lesion Segmentation Tool, LST). During this procedure, lesions were seeded according to spatial and intensity probabilities on T1 images and hypersignal outliers on T2FLAIR images. The initial threshold was set at 0.3 to create a conserved binary lesion map from which the growth algorithm (Schmidt et al., 2019) expanded these seeds to grow lesion probability maps (LPM) along high-signal T2FLastivoid growth. The LPM is then visually examined against T2FLAIR images to ensure accurate capture of WMH volum. Figure 2 shows the results of white matter segmentation of iNPH patients using LST toolbox. The WMH is the yellow region marked in the figure.

### 2.5. Method of statistics

SPSS 26.0 statistical software and GraphPad9.3 version were used to complete all data analysis and statistical charts. The Shapiro–Wilk test was used for the normality test because of the small sample size. The differences between the groups were compared. When the samples met the standard normal distribution, two independent sample t-tests were used. Two independent sample non-parametric tests (Mann–Whitney *U* test) were used when the samples did not meet the standard normal distributions. Three groups comparison were performed using one-way ANOVA with a Tukey’s multiple comparisons test. We reported actual values of p from the ANOVA. Linear regression was used to examine the relationship between gait parameters and regional WMH and cerebrospinal fluid markers in iNPH patients. The mediation analysis was used for further verification, and the mediation effect model was built with regional WMH as the mediator variable, NfL as the independent variable, and gait parameter as the dependent variable to clarify whether the relationship between the independent variable and the dependent variable was affected by the intermediary variable. For the missing value problem, we first identify the predictor of the missing variable. Then the prediction equation is generated using the no-miss record, and the missing value is predicted. Values of *p* < 0.05 were considered statistically significant.

## 3. Results

### 3.1. Demographic variables and clinical assessment

We included 48 patients with iNPH and 20 normal older individuals. Men accounted for 58% of iNPH patients and 60% of normal older individuals. There was no significant difference in age (*p* = 0.23, Table 1), iNPH comorbidities of hypertension (*p* = 0.59, Table 1), and diabetes (*p* = 0.71, Table 1) between the two groups and the control group. There were statistical differences in cerebrospinal fluid markers Aβ42, t-tau, and p-tau between the two groups, and iNHP patients were lower than normal older individuals. There was also a statistical difference in NfL in CSF between the two groups, with higher levels of NfL in CSF in patients with iNHP. As for the WMH, there were statistical differences between the two groups in the volume of WMH in the frontal lobe, parietal lobe, temporal lobe, occipital lobe, basal ganglia region, periventricular and deep part of the brain (*p* < 0.05, Table 1), and iNPH patients were all higher than the normal older individuals.

**Table 1:** Demographic data, preoperative symptoms, and protein concentrations.

Characteristic	iNPH	Control	95%CI	T/Z	value of *p*
Total number	48	20	–	–	–
Age, years, mean (range)	76.94 ± 6.49	73.95 ± 8.17	(−0.85, 7.07)	2.54	0.23^a^
Male, n (%)	28(58%)	12(60%)	–	0.14	0.82^b^
t-tau	138.23 ± 6.78	150.66 ± 6.09	(−16.48, −9.19)	5.62	0.028^a^
p-tau	41.78 ± 5.84	54.77 ± 6.91	(−17.33, −7.56)	5.09	0.02^a^
Aβ42	188.42 ± 73.62	656.92 ± 56.98	(−487.37, −449.28)	4.74	0.033^a^
NfL	1610.32 ± 290.38	764.19 ± 201.55	(795.37, 897.02)	9.88	0.002^a^
Comorbidity, n (%)	–	–	–		–
Diabetes	23(48%)	9(45%)	–	0.23	0.71^b^
Hypertension	15(31%)	7(35%)	–	0.64	0.59^b^
Previous stroke (>3 months)	12(25%)	–	–	–	–
WMH location (mL)	–	–	–	–	–
Frontal	21.98 ± 5.00	4.67 ± 2.89	(15.47, 19.07)	6.14	0.016^a^
Parietal	8.90 ± 2.06	1.62 ± 0.75	(6.50, 8.77)	13.42	0.000^a^
Temporal	3.10 ± 2.30	2.09 ± 1.78	(0.98, 1.39)	6.86	0.011^a^
Occipital	2.06 ± 0.42	0.92 ± 0.32	(0.92, 1.36)	10.37	0.001^a^
PVH	27.09 ± 7.83	6.78 ± 1.84	(17.89, 23.97)	13.74	0.000^a^
DWMH	1.41 ± 0.21	0.49 ± 0.29	(0.91, 1.31)	5.14	0.027^a^
iNPHGS	7.06 ± 2.21	–	–	–	–
mRS	3.44 ± 0.87	–	–	–	–
MMSE	22.67 ± 3.40	27.25 ± 1.36	(−6.13, −3.79)	9.60	0.000^a^
3 m-TUG	46.62 ± 26.43	–	–		–
10 m speed (m/s)	52.44 ± 27.97	–	–		–
Urinary	1.32 ± 1.24	–	–		–

^a^Two-sample *t*-test; ^b^nonparametric test (Mann–Whitney *U* test). *p* < 0.05 was considered significant. iNPH, idiopathic normal pressure hydrocephalus; NfL, neurofilament light chain; WMH, White matter hyperintensity; PVH, periventricular white matter hyperintensity; DWMH, deep white matter hyperintensities; iNPHGS, iNPH grading scale; TUG, time up & go; MMSE, MMSE, Mini-Mental State Examination.

### 3.2. Association of cerebrospinal fluid markers and regional WMH with iNPH gait disorder

A univariate linear regression model was used to evaluate the relationship among CSF markers, regional WMH volume, and gait parameters in patients with iNPH. Among cerebrospinal fluid markers, NfL level was linearly correlated with the time and number of steps in both the 3 m-TUG and the 10 m straight walk test (*p* < 0.05, Table 2). In the regional WMH, there was a linear correlation between frontal WMH volume, PVH, and the time and number of steps in the 3 m-TUG test and the 10 m straight walking test (*p* < 0.05, Table 2). However, there was a linear correlation between the volume of WMH in the parietal lobe and the time and number of steps in the 3 m-TUG test (*p* < 0.05, Table 2). There was no linear correlation in the 10 m straight walking test (*p* > 0.05, Table 2).

**Table 2:** Linear regression model showed the influence of each CSF pathological marker and regional WMH on gait disorder.

Measure	3-TUG time			3-TUG steeps			10 m time			10 m steeps			T	*p*	95%CI	T	*p*	95%CI	T	*p*	95%CI	T	*p*	95%CI
NfL	4.57	0.000	(0.03, 0.07)	4.37	0.000	(8.03, 20.90)	3.73	0.001	(0.03, 0.10)	3.44	0.001	(0.02, 0.09)
Aβ	0.92	0.36	(−0.25, 0.09)	1.37	0.18	(−0.80, 0.16)	0.71	0.48	(−0.60, 0.29)	0.45	0.65	(−0.46, 0.29)
Frontal lobe WMH	6.35	0.000	(0.84, 1.59)	6.225	0.000	(0.34, 0.66)	5.3	0.000	(2.49, 5.70)	3.99	0.000	(1.47, 4.47)
Parietal lobe WMH	3.34	0.002	(0.49, 1.89)	2.34	0.020	(0.11,1.30)	0.29	0.772	(−3.23, 4.38)	1.55	0.129	(−0.74, 5.65)
PVH	5.09	0.000	(0.42, 0.94)	4.74	0.000	(0.37, 0.91)	2.52	0.015	(0.47, 3.19)	6.77	0.000	(1.99, 3.73)

NfL, neurofilament light; WMH, white matter hyperintensities; TUG, time up & go.

### 3.3. Association between gait disorders and cerebrospinal fluid markers

**Figure 3:**
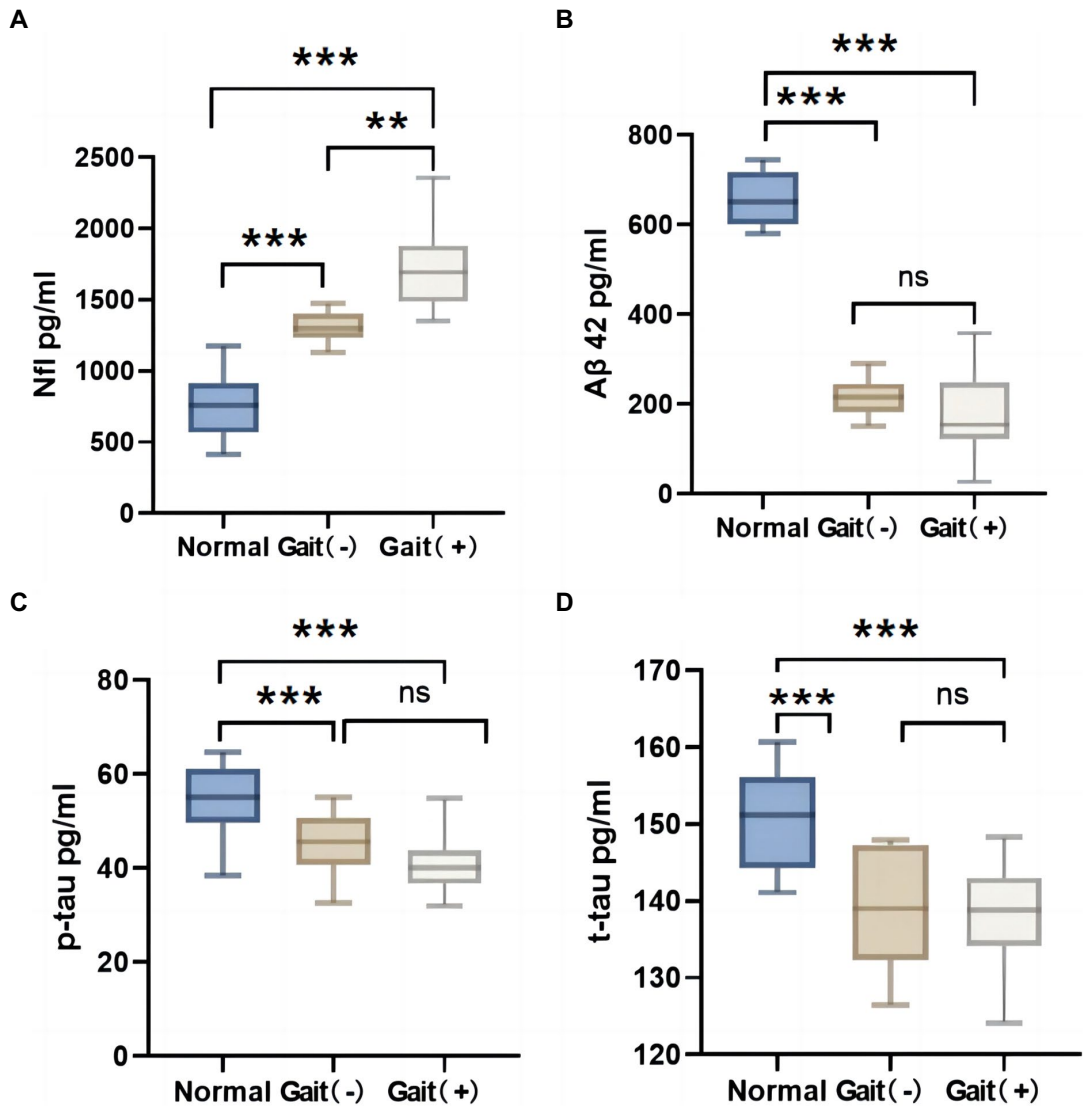
Difference of CSF markers in normal elderly, gait (+) group and gait (−) group. **(A)** Nfl in CSF of the gait (+) group was higher than that of the gait (−) group and the normal olders group. **(B–D)** Aβ42, p-tau and t-tau in CSF of the gait (+) group were lower than those of the normal olders group. And there was no statistical difference between the gait (+) group and gait (−) group. ***p < 0.001, **p < 0.01, and *p < 0.05. NfL, neurofilament light.

The iNPH patients were categorized into a gait (+) impairment group and a non-impairment gait (−) group according to the iNPHGS scale gait score. There were no significant differences in age, hypertension, diabetes and MMSE scores between the gait (+) group and the gait (−) group (Table 3, *p* > 0.05). By comparison with normal older individuals, the level of NfL in CSF of iNPH patients was higher than that of normal older individuals (Figure 3A, *p* < 0.05), and the level of Aβ42, t-tau, and P-tau were lower than that of normal older individuals (Figures 3B–D, *p* < 0.05). Gait (+) and gait (−) have A higher NfL level in the former than in the latter, and no significant differences in Aβ42, t-tau, and P-tau levels between the two groups (p > 0.05).

**Table 3:** Differences in baseline data between the two subgroups gait (+) versus gait (−) and improvement versus no improvement.

	Gait (+) (35)	Gait (−) (13)	95%CI	T/Z	Value of *p*	Improvement (24)	No improvement (11)	95%CI	T/Z	Value of *p*
Age	77.69 ± 4.43	74.92 ± 4.56	(−0.16, 5.68)	1.91	0.740^a^	77.79 ± 4.79	77.45 ± 3.74	(−2.99, 3.67)	0.21	0.310^a^
Diabetes	40%	31%	–	0.10	0.924^b^	38%	45%	–	0.53	0.660^b^
Hypertension	34%	36%	–	0.41	0.690^b^	33%	36%	–	0.23	0.863^b^
MMSE	22.46 ± 2.98	23.23 ± 2.49	(−2.65, 1.10)	0.83	0.671^a^	23.46 ± 2.72	20.27 ± 2.37	(−3.35, 1.34)	1.34	0.743^a^

^a^Two-sample *t*-test; ^b^nonparametric test (Mann–Whitney *U* test). *p* < 0.05 was considered significant.

### 3.4. Association between gait disturbance and high regional WMH

**Figure 4:**
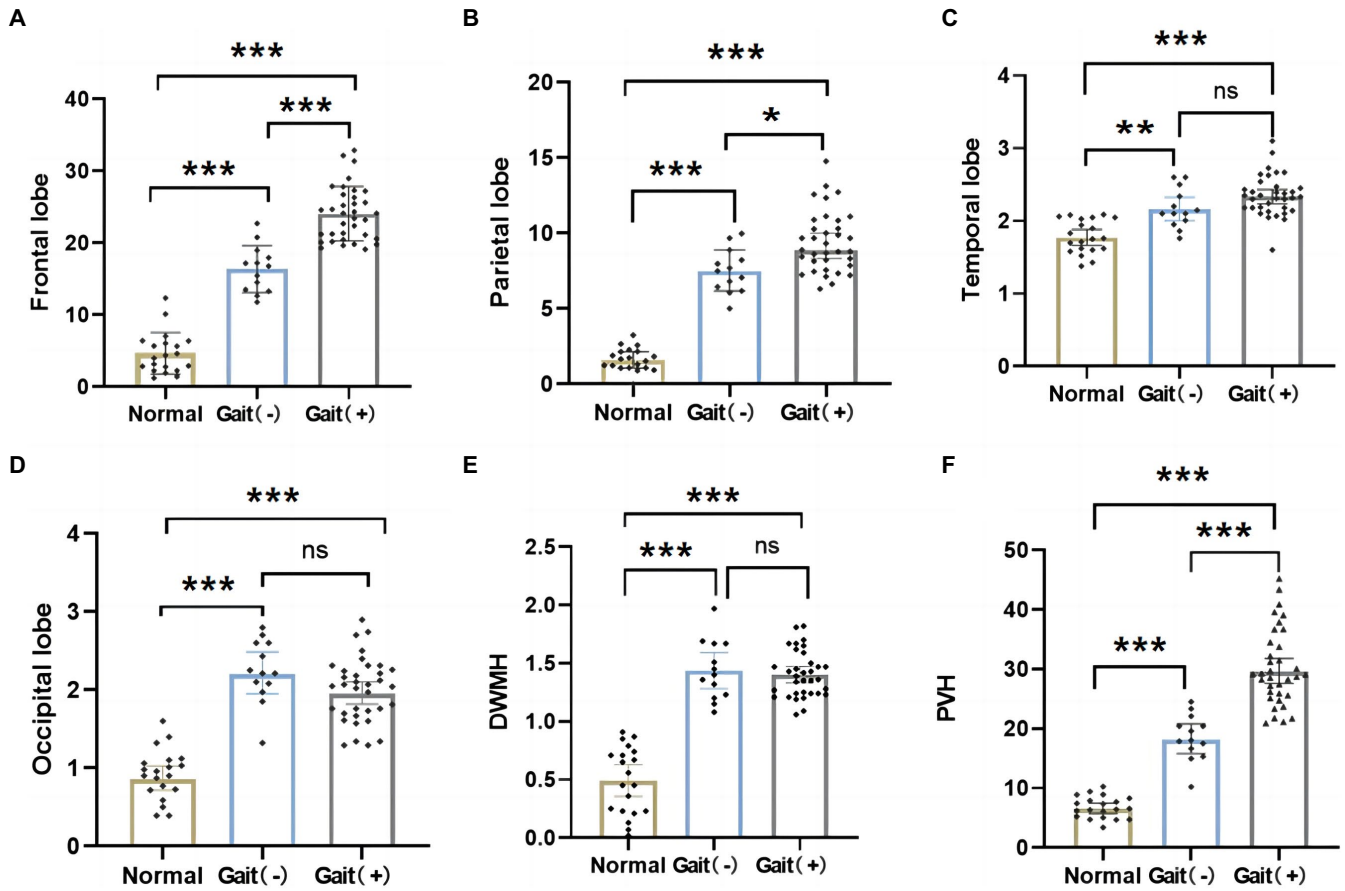
Differences in regional WMH among normal olders, gait (+) group and gait (−) group. **(A,B,F)** WMH in the frontal and parietal lobes, PVH of the gait (+) group were higher than those in the gait (−) group and the normal olders. **(C–E)** WMH in temporal and occipital lobes, DWMH were higher in the gait (+) group than the normal olders. And there was no statistical difference between the gait (+) group and gait (−) group. ***p < 0.001, **p < 0.01, and *p < 0.05. PVH, periventricular white matter hyperintensity; DWMH, deep white matter hyperintensities.

When the differences in WMH volume between the three groups were compared, it was discovered that the WMH volume in all brain regions of iNPH patients was higher than that of normal older people (Figures 4A–F, *p* > 0.05). The gait (+) group in the WMH volume in the frontal–parietal lobe and PVH was higher than that in the gait (−) group (Figures 4A–C, *p* > 0.05). However, there was no significant difference in the WMH volume in the temporal lobe, basal ganglia, and deep (Figures 4D–F, *p* > 0.05).

### 3.5. Results of mediating effect among NfL, frontal and periventricular WMH and gait disorder in iNPH patients

**Figure 5:**
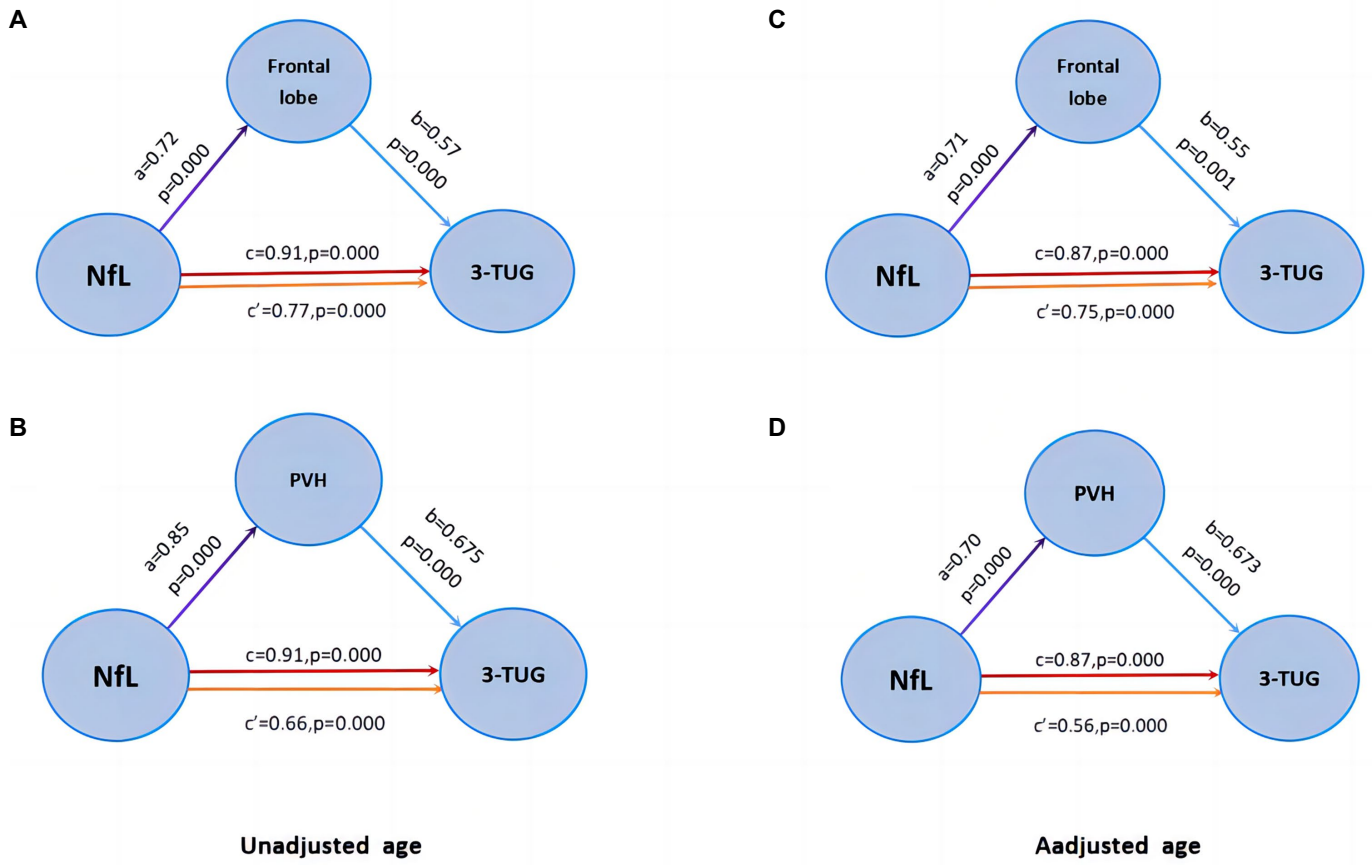
Mediation effect model. **(A–B)** Frontal WMH and PVH partially mediate the relationship between Nfl and gait disorder. **(C–D)** There was still a statistical difference after adjusting for age.

The mediation effect model was constructed in the gait (+) group patients. We found that the 3 m-TUG time parameter was positively correlated with frontal WMH volume (Figure 5A, *b* = 0.57, *p* = 0.000) and periventricular WMH volume (Figure 5B, *b* = 0.675, *p* = 0.000), NfL was positively correlated with frontal WMH volume (Figure 5A, *a* = 0.72, *p* = 0.000) was positively correlated with periventricular WMH volume (Figure 5B, *a* = 0.85, *p* = 0.000). The regional WMH volume was used as the mediating variable, the NfL concentration as the independent variable, and the 3 m-TUG time parameter as the dependent variable. Mediating effects showed that NfL may partially mediate the occurrence of gait disorders in iNPH patients through frontal and periventricular white matter lesions (Figures 5A,B, c′coefficient is still significant but less than c). There was still a statistical difference after adjusting for age (Figures 5C,D).

### 3.6. Association of high regional WMH with NfL and 1-year prognosis after shunt surgery

**Figure 6:**
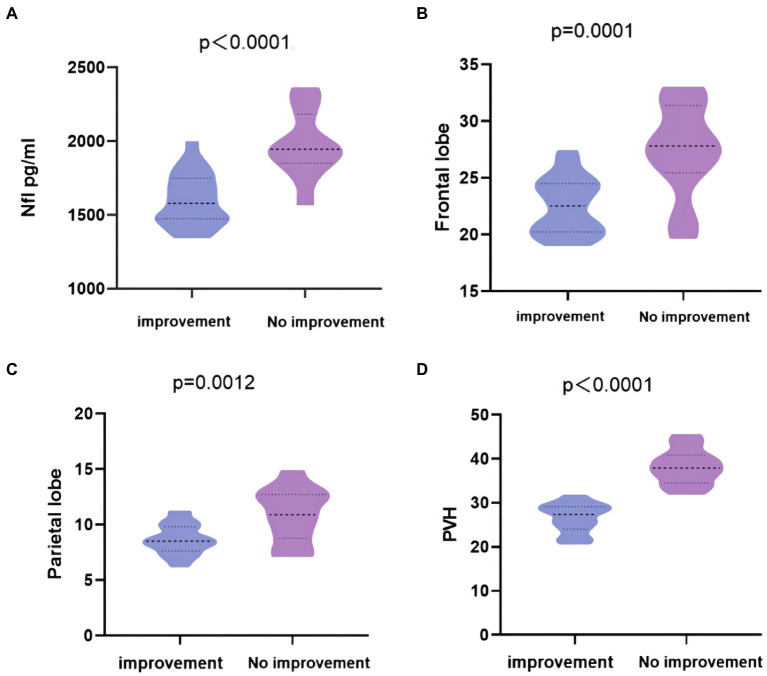
Differences in regional WMH and NfL between improvement group and no improvement group in iNPH with gait disorder 1 year after surgery. **(A)** The concentration of CSF Nfl in the improvement group was lower than no improvement group. **(B–D)** WMH in the frontal and parietal lobes, PVH of the improvement group were lower than no improvement group. All of the above have statistical differences.

Through 1-year follow-up of the patients after the bypass surgery, it was found that 24 of the 35 patients in the gait (+) group improved, while 11 had no significant improvement. There were no significant differences in age, hypertension, diabetes and MMSE scores between the improvement group and the no improvement group (Table 3, *p* > 0.05). Comparing cerebrospinal fluid marker levels between the two groups showed that the level of cerebrospinal fluid NfL in the improvement group was lower than that in the no improvement group (Figure 6A, *p* < 0.0001, *t* = 6.09). The comparison results of WMH volume between the two groups showed that WMH volume and PVH in the frontal–parietal lobe of the improvement group were lower than those of the no improvement group (Figure 6B, *p* = 0.0001, *t* = 4.18; Figure 6C, *p* = 0.0012, *t* = 3.58; Figure 6D, *p* < 0.0001, *t* = 5.27).

## 4. Discussion

This study is the first to include iNPH lobular WMH volume to study the relationship between WMH body and gait disorder and further investigate the role of CSF markers in this. Our results showed that compared with normal older individuals, the volume of WMH in the frontoparietemporal lobe, basal ganglia region, periventricular and deep part of the brain in iNPH patients was higher than that in normal older individuals. In CSF markers, the levels of t-tau, p-tau, and Aβ42 were lower than those in the control group, while the levels of NfL were higher than those in the control group. Our main results showed that WMH volume and PVH in the frontal–parietal lobe of patients with iNPH gait disorder were correlated with gait disorder. The more moderate the lesions were in the frontal–parietal lobe, and periventricular white matter, the more obvious the gait disorder was in iNPH patients. In iNPH patients, NfL level was associated with gait disorder concerning cerebrospinal fluid markers. The higher the level of NfL, the more significant the gait disorder in patients with iNPH. In a 1-year follow-up of iNPH patients after shunt surgery, we found that WMH volume and CSF NfL levels in the frontal–parietal and periventricular lobes were associated with postoperative improvement in gait disorder.

Nearly half of iNPH patients showed AD-related Aβ lesions on biopsy, and 10% showed both Aβ and tau pathology (Ingelsson et al., 2004; Leinonen et al., 2010). In A meta-analysis, Chen et al. (2017) evaluated cerebrospinal fluid Aβ42, t-tau, and p-tau in patients with iNPH by including 10 studies. The study included 413 patients with iNPH, 186 patients with AD, and 147 healthy controls. Results showed that the Aβ42, t-tau, and p-tau levels were significantly reduced in patients with iNPH compared with the control group. This study’s results are consistent with our findings. Current explanatory theories for reduced CSF biomarker levels in iNPH include reduced periventricular metabolism (Momjian et al., 2004; Jeppsson et al., 2013) and reduced flow of metabolites from tissue fluid to CSF, for example, through lymphatic system damage (Iliff et al., 2013; Graff-Radford, 2014; Eide and Ringstad, 2019) or dilution of fixed amounts of biomarkers (Jingami et al., 2015; Vanninen et al., 2021). However, this is consistent with previous PET and MRI studies on iNPH. Another possible explanation is that retrograde CSF dynamics in patients with iNPH lead to decreased extracellular fluid flow to the heart and decreased extracellular fluid clearance, and decreased metabolism may also be secondary to changes in CSF dynamics (Jeppsson et al., 2013). The widespread degradation of APP-derived proteins in iNPH cannot be ruled out.

There are few studies on gait disorder and WMH in iNPH patients. Our results show that parietal WMH and periventricular WMH may be related to gait disorders in patients with iNPH. At present, there are studies through the use of diffusion tensor imaging detection of water molecules to reflect the direction of the white matter conduction beam and the detection of water molecules in the area of interest with anisotropy fraction Fractional anisotropy, FA value quantification, to study white matter lesions or different parts of the conduction beam compression degree. Koyama et al. (2012) significantly increased the frontal FA value of corticospinal tract and corpus callosum radiation. Hattori et al. (2012) also obtained the same result in the conduction bundle specificity analysis of iNPH patients. Tang et al. (2021) showed that iNPH patients with more WMH had poorer gait performance, and fiber bundles were most significantly correlated with gait index. In patients with iNPH, the white matter FA value in the prefrontal area is decreased. The movement symptoms of iNPH may be related to glial cell proliferation and nerve fiber damage in this area because of the passage of some nerve fibers in the functional corticostriatal circuit (including the higher motor circuit; Lenfeldt et al., 2011).

Aoki et al. (2013) found and reached the following views: The cortical areas involved in the gait function of iNPH patients include the left frontal motor cortex of gait state, the auxiliary motor area of gait speed (SMA), the left dorsolateral prefrontal cortex (DLPFC) and the right prefrontal cortex, which may be caused by the shear force generated by the change of cerebrospinal fluid flow that destroys the sensory-motor in the brain. Previous studies have shown that gait disorder is related to structural changes in white matter and functional changes in interhemispheric, frontal, temporal, medial, and parietal connections (Khoo et al., 2016). Griffa et al. (2020) found that white matter features in the periventricular, frontal lobe and temporal brain circuits of iNPH patients differed from those in healthy older people by studying the structural connections in the brain iNPH patients. Studies have also indicated that the frontal–parietal subcortical cerebellar circuit may be a vulnerable area for the pathophysiological mechanism of iNPH (Griffa et al., 2020). In addition, gait disorders are also associated with dysfunction of gabaergic and cholinergic inhibitory circuits in the frontal motor cortex (Chistyakov et al., 2012; Nardone et al., 2019). The more severe the lesion of the white matter structure, the neurotransmitter transmission of the motor cortex circuit is blocked, thus affecting gait. Therefore, in our study, we propose that changes in the white matter structure of the frontal–parietal lobe and surrounding ventricles may induce the disrupted transmission of information related to abnormal gait.

NfL in CSF is a marker of myelin axonal degeneration and is elevated in neurodegenerative, neuroinflammatory, traumatic, and cerebrovascular diseases (Gaetani et al., 2019). There are few studies on WMH and NfL in patients with iNP. In a study of DTI assessment in normal older individuals, NfL levels in CSF were associated with white matter integrity and WMH but not amyloid pathology (Moore et al., 2018). Meeker et al. showed in their study on the relationship between NfL and aging that elevated NfL in cerebrospinal fluid was associated with greater total WMH volume (Meeker et al., 2022). NfL is an axon structural protein, and WMH reflects demyelination and axon loss, so it is not surprising that it is most closely related to WMH. In a large sample study, Braun et al. reported that NfL levels in CSF were associated with adverse outcomes in patients with iNPH, which may be attributed to more damage to white matter structures (Malmberg et al., 2017).

Our results show that the level of NfL in CSF in patients with iNPH is higher than that in healthy older adults and is associated with gait disorders in patients. Mediated analysis showed that NfL might affect the gait of patients with iNPH through frontal–parietal and periventricular WMH. Previous studies have found that preoperative NfL level is correlated with the severity of clinical symptoms (Tullberg et al., 1998; Agren-Wilsson et al., 2007; Tullberg et al., 2008). In addition, in our longitudinal follow-up, patients with high NfL levels did not improve significantly 1 year after surgery. Our severe results showed that patients without improvement also had more serious white matter lesions in the frontal–parietal lobe and periventricular. Does this mean that patients with severe white matter lesions in these areas should not undergo shunt surgery in preoperative evaluation? In a recent paper, researchers give us a negative answer. The results showed that although PVH was associated with adverse outcomes after shunt surgery, there was no statistical difference in PVH between shunt responders and non-responders (Snöbohm et al., 2022). The results were not consistent with our findings. However, this may be related to sample size, patient age, and cerebrovascular risk factors. In conclusion, NfL levels in CSF reflect white matter integrity, and the structural integrity of white matter fibers plays an essential role in transmitting information related to subcortical gait.

### 4.1. Limitations of the study

There are some limitations to our study. First, the sample size of this study is relatively small, which may lead to results bias. Further expansion of sample size or even multi-center verification is needed. There were no significant differences in age or cardiovascular risk factors between iNPH patients and controls. However, previous studies showed that WMH is associated with age and other cardiovascular factors such as hypertension and diabetes. Therefore, in addition to controlling for age accidents, cardiovascular and cerebrovascular risk factors should also be controlled to make the results more reliable. This is also a limitation of our research. In addition, in the follow-up analysis of this study, the numbers of the two groups with and without improvement were too small, which weakened the reliability of the research conclusions. Secondly, the study on the correlation between regional WMH volume, CSF markers, and gait disorders was cross-sectional. Therefore, our study lacked longitudinal analysis of imaging changes and cerebrospinal fluid changes before and after iNPH. Finally, our study did not calculate the volume of WMH in the subtentorial areas, such as the cerebellum and brainstem, which may also be related to the gait disorder of iNPH patients. Our study may have ignored this part. These will be the focus of our future research directions.

## 5. Conclusion

In summary, we associated regional WMH volume, NfL level in CSF, and gait disorder in patients with iNPH. The results showed that WMH in the frontal–parietal lobe and periventricular area of iNPH patients might be related to the occurrence of gait disorder. The NfL level of CSF was significantly higher in iNPH patients, which was also associated with gait disorder. The final mediating analysis shows that NfL may play a potential role in the occurrence of iNPH gait disorder through WMH. Therefore, this provides some references for exploring the pathogenesis of iNPH symptoms.

## Data availability statement

The original contributions presented in the study are included in the article/supplementary material, further inquiries can be directed to the corresponding authors.

## Ethics statement

The studies involving human participants were reviewed and approved by the Ethical Committee of Huadong Hospital of Fudan University. The patients/participants provided their written informed consent to participate in this study. Written informed consent was obtained from the individual(s) for the publication of any potentially identifiable images or data included in this article.

## Author contributions

LY and FY designed the study. LY performed the research and wrote the initial draft of the manuscript. XF analyzed the data. YD, WW, and AY contributed to refining the ideas, carrying out additional analyses and finalizing this manuscript. All authors contributed to the article and approved the submitted version.

## Funding

This work was supported by the Shanghai Municipal Key Clinical Specialty (grant number: shslczdzk02801), the Shanghai Municipal Health Commission (grant number: 2020YJZX0109), Shanghai Sailing Program (grant number: 21YF1411600), and the Scientific Research Foundation of Shanghai Municipal Commission of Health and Family Planning (201740003, 20184Y0098). The funder had no role in study design, data analysis, publication decision, or manuscript preparation.

## Conflict of interest

The authors declare that the research was conducted in the absence of any commercial or financial relationships that could be construed as a potential conflict of interest.

## Publisher’s note

All claims expressed in this article are solely those of the authors and do not necessarily represent those of their affiliated organizations, or those of the publisher, the editors and the reviewers. Any product that may be evaluated in this article, or claim that may be made by its manufacturer, is not guaranteed or endorsed by the publisher.

## Abbreviations

iNPH: idiopathic normal pressure hydrocephalus
CSF: cerebrospinal fluid
FLAIR: fluid-attenuated inversion recovery
PVH: periventricular white matter hyperintensity
T1WI: longitudinal relaxation-weighted images
T2WI: transverse relaxation-weighted images
DWI: diffusion-weighted images
DWMH: deep white matter hyperintensities
LPM: lesion probability maps
DLPFC: dorsolateral prefrontal cortex
TUG: time up & go
iNPHGS: iNPH grading scale
LST: Lesion Segmentation Tool
